# Lack of transient receptor potential vanilloid 1 channel modulates the development of neurogenic bladder dysfunction induced by cross-sensitization in afferent pathways

**DOI:** 10.1186/1742-2094-10-3

**Published:** 2013-01-11

**Authors:** Qi Lei, Xiao-Qing Pan, Antonio N Villamor, Tirsit S Asfaw, Shaohua Chang, Steven A Zderic, Anna P Malykhina

**Affiliations:** 1 Department of Surgery, Division of Urology, University of Pennsylvania, 500 South Ridgeway Avenue, Glenolden, PA, 19036, USA; 2 Department of Obstetrics and Gynecology, Weill Cornell Medical College, 1305 York Avenue, New York, NY, 10065, USA; 3 Department of Surgery, Cooper University, One Cooper Plaza, Camden, NJ, 08103, USA; 4 John W. Duckett Center for Pediatric Urology, Children’s Hospital of Philadelphia, 34th Street and Civic Center Blvd, Philadelphia, PA, 19104, USA

**Keywords:** Chronic pelvic pain, Bladder sensory neurons, Neurogenic bladder, Detrusor contractility

## Abstract

**Background:**

Bladder pain of unknown etiology has been associated with co-morbid conditions and functional abnormalities in neighboring pelvic organs. Mechanisms underlying pain co-morbidities include cross-sensitization, which occurs predominantly via convergent neural pathways connecting distinct pelvic organs. Our previous results showed that colonic inflammation caused detrusor instability via activation of transient receptor potential vanilloid 1 (TRPV1) signaling pathways, therefore, we aimed to determine whether neurogenic bladder dysfunction can develop in the absence of TRPV1 receptors.

**Methods:**

Adult male C57BL/6 wild-type (WT) and TRPV1^−/−^ (knockout) mice were used in this study. Colonic inflammation was induced by intracolonic trinitrobenzene sulfonic acid (TNBS). The effects of transient colitis on abdominal sensitivity and function of the urinary bladder were evaluated by cystometry, contractility and relaxation of detrusor smooth muscle (DSM) *in vitro* to various stimuli, gene and protein expression of voltage-gated sodium channels in bladder sensory neurons, and pelvic responses to mechanical stimulation.

**Results:**

Knockout of TRPV1 gene did not eliminate the development of cross-sensitization between the colon and urinary bladder. However, TRPV1^−/−^ mice had prolonged intermicturition interval and increased number of non-voiding contractions at baseline followed by reduced urodynamic responses during active colitis. Contractility of DSM was up-regulated in response to KCl in TRPV1^−/−^ mice with inflamed colon. Application of Rho-kinase inhibitor caused relaxation of DSM in WT but not in TRPV1^−/−^ mice during colonic inflammation. TRPV1^−/−^ mice demonstrated blunted effects of TNBS-induced colitis on expression and function of voltage-gated sodium channels in bladder sensory neurons, and delayed development of abdominal hypersensitivity upon colon-bladder cross-talk in genetically modified animals.

**Conclusions:**

The lack of TRPV1 receptors does not eliminate the development of cross-sensitization in the pelvis. However, the function of the urinary bladder significantly differs between WT and TRPV^−/−^ mice especially upon development of colon-bladder cross-sensitization induced by transient colitis. Our results suggest that TRPV1 pathways may participate in the development of chronic pelvic pain co-morbidities in humans.

## Background

Chronic pelvic pain (CPP) is a common symptom of many urologic and gastrointestinal disorders, including interstitial cystitis/bladder pain syndrome (IC/BPS), irritable bowel syndrome (IBS), and non-bacterial prostatitis/chronic pelvic pain syndrome. A high level of co-morbidities among CPP disorders is well documented in the clinical setting [1-3]. The etiology of IC/BPS is often complicated by visceral hypersensitivity arising from the gastrointestinal (GI) tract [1,4]. Similarly, a significant number of patients with IBS complain of urinary symptoms, including nocturia [5], frequency and urgency of micturition [2] and incomplete bladder emptying [6]. Viscero-visceral reflexes between the lower GI and urinary tracts are controlled by both autonomic and central nervous systems (CNS), suggesting the dominant role of neural pathways in pelvic organ co-morbidities.

The latest research efforts aimed at understanding the mechanisms underlying complex CPP disorders provided evidence that cross-sensitization in afferent pathways may initiate the development of neurogenic inflammation in the pelvis and functional chronic pelvic pain [7,8]. Cross-sensitization among pelvic organs implies the transmission of noxious stimuli from a diseased pelvic organ to an adjacent normal structure resulting in the occurrence of functional changes in the latter [9]. A pathological condition developed in one of the pelvic organs may cause initial sensitization of peripheral afferent fibers and sensory neurons. These primary changes then lead to amplification of nociceptive signaling in the CNS followed by descending modulatory input from the CNS to the periphery [8-10].

The use of animal models provides insight into investigation of functional changes in nerve fibers and neurons as research of these in humans has certain challenges. Of major interest for the studies of functional co-morbidities are animal models in which an initial acute stimulus (inflammation, infection, noxious distension, trauma, and so on) is transient but powerful enough to cause long-lasting abdominal hypersensitivity and hyperexcitability of visceral afferents [11-13]. Several independent investigations established that transient inflammation of the distal colon in animal models induces the occurrence of neurogenic cystitis due to cross-sensitization in neural pathways [14-19]. After recovery from transient colitis, neither the colon nor the urinary bladder demonstrated detectable histological/biochemical changes suggestive of active inflammation. However, the bladder develops many signs of neurogenic dysfunction evaluated by cystometry [14], hyperexcitability of bladder sensory [13,16] and spinal [20] neurons, release of pro-inflammatory neuropeptides in the bladder [21], and altered detrusor contractility [14,22].

Recent functional and molecular studies from our laboratory identified the changes in a number of nociception-related genes and an increased release of pro-inflammatory neuropeptides in the urinary bladder following transient inflammation of the distal gut [16,21]. We also established that these changes are associated with activation of intracolonic transient receptor potential vanilloid 1 (TRPV1) receptors [21]. TRPV1 is a non-specific cation channel activated by heat, protons, and vanilloids [23]. It is expressed in many visceral organs including the colon and urinary bladder [24,25] with the highest level of expression in primary sensory neurons innervating the visceral and somatic structures [23]. A growing body of evidence has led to the emergence of TRPV1 as a key player in sensory transduction. The role of TRPV1 in somatic pain has been extensively studied during the last decade, however, much less is known about the involvement of TRPV1 in viscero-visceral hyperalgesia and chronic visceral pain. To address the role of TRPV1 in nociceptive signal transmission from the inflamed colon to the urinary bladder and development of neurogenic bladder dysfunction, we evaluated the effects of TRPV1 gene knockout on the function of the urinary bladder, detrusor contractility and associated signaling *in vivo* and *in vitro* in a model of colon-bladder cross-sensitization using TRPV1 knockout mice.

## Materials and methods

### Animals and experimental groups

Adult male C57BL/6 wild-type (WT) and TRPV1 gene-deleted (TRPV1^−/−^, KO) mice (10 to 12 wks, 20 to 25 g, Jackson Laboratories, Bar Harbor, ME, USA; N = 56) were used in this study. Animals were housed in a regulated environment, with free access to food and water and maintained on a 12:12-h light/dark cycle. There were no overt differences in feeding behavior, litter size, growth rate and body weight between the WT and TRPV1^−/−^ mice. Animals were divided into four experimental groups: 1) WT control group; 2) WT group with colonic inflammation; 3) TRPV1^−/−^ control group; and 4) TRPV1^−/−^ mice with colonic inflammation. Animals from each group were used for *in vivo* and *in vitro* experiments at 3 to 5 days after intracolonic treatments. All protocols were approved by the University of Pennsylvania Institutional Animal Care and Use Committee and adhered to the guidelines for experimental pain in animals published by the International Association for the Study of Pain.

### Animal model of colon-bladder cross-sensitization induced by colonic inflammation

Transient colonic inflammation was induced by a single administration of 2,4,6-trinitrobenzene sulfonic acid (TNBS, 60 mg/kg, in 25% C_2_H_5_OH), a chemical irritant that causes inflammation in the intestine. The TNBS solution was prepared fresh before the instillation procedure. Animals were anesthetized with isoflurane (VEDCO Inc., St. Joseph, MO, USA) and intracolonic treatments were performed via a flexible catheter connected to a 1 ml syringe. Mice received 0.3 ml of either vehicle (25% ethanol, control groups) or TNBS (inflammation groups) solution. To assess the severity of induced colonic inflammation *in vivo*, the daily disease activity index (DAI) was calculated by scoring changes in animal weight, occult blood positivity, gross bleeding, and stool consistency as previously described [16].

### Histological and biochemical evaluation of inflammation *in vitro*

Segments of the colon and the entire urinary bladder were isolated from WT and TRPV1^−/−^ mice at the end of physiological experiments. One part of each tissue was fixed in 4% paraformaldehyde for histological evaluation. Fixed samples of the colon and urinary bladder were embedded in paraffin and sectioned at 10 μm thickness. The samples were stained with hematoxylin and eosin (H&E staining kit, Richard-Allan Scientific, Kalamazoo, MI, USA) and assessed for the signs of inflammation under a light microscope. Another part of each specimen was snap-frozen in liquid nitrogen and stored at −80°C for running a myeloperoxidase (MPO) assay using MPO ELISA kit (Alpco, Salem, NH, USA) as previously described [16].

### Surgical procedure to catheterize the urinary bladder for cystometry

Mice included in the groups for urodynamic evaluation of the urinary bladder function (awake cystometry) underwent survival surgical procedure for catheter insertion. An animal was anesthetized with isoflurane (VEDCO, St. Joseph, MO, USA), and a polytetrafluoroethylene catheter with a blunted end (Catamount Research, St. Albans, VT, USA) was sutured in place at the bladder dome and tunneled out the abdomen to the nape of the neck where it was then inserted into the end of a 22-gauge angiocath intravenous catheter. Upon determination of the optimal length, the catheter was affixed to the angiocath with super glue. The angiocath was first tested with a gentle saline infusion to reveal no leak at the bladder, then capped and the abdomen was closed in layers. The angiocath was anchored to the fascia and skin of the neck using two to three 3–0 Vicryl sutures. After recovery from anesthesia, animals were returned to the animal facility and kept in individual cages to avoid possible damage to the catheters by their cage mates. Mice were allowed to recover from surgery for 4 days followed by cystometric evaluation of bladder function under normal physiological conditions without anesthesia (baseline cystometry). After initial urodynamic evaluation, mice received a single intracolonic instillation of either vehicle or TNBS, as described above, followed by repeated cystometric evaluation at 3 to 5 days post-treatment.

### Assessment of urodynamic parameters of bladder function

Conscious mice were placed in cystometry cages (16 cm width, 12 cm height, and 24 cm length) without any restraint and allowed to acclimate for 30 min. The tip of the exteriorized bladder catheter located at the base of the mouse neck was connected to a pressure transducer and an infusion pump of the cystometry station (Small Animal Laboratory Cystometry, Catamount Research and Development, St. Albans, VT, USA) using a T-shaped valve. Room temperature saline solution (0.9% NaCl) was infused in the bladder at a rate of 10 μl/min. Voided urine was collected in the tray connected to a force displacement transducer integrated into the data acquisition system. Each animal was observed for up to six to eight voiding cycles. Urodynamic values were recorded continuously using data acquisition software (Small Animal Laboratory Cystometry, Catamount Research and Development, St. Albans, VT, USA). The following cystometric parameters were recorded and analyzed in this study: bladder capacity, pressure at the start of micturition, micturition rate, intravesical pressure, inter-micturition interval, and the number of non-micturition contractions. Non-micturition contractions were defined as increased values in detrusor pressure from baseline that had amplitudes of at least a third of maximal pressure at the start of micturition. Cystometric parameters were uploaded from the acquisition software into analysis software (SOF-552 Cystometry Data Analysis, Version 1.4, Catamount Research and Development Inc., St. Albans, VT, USA) for statistical analysis.

### *In vitro* measurements of detrusor contractility

For *in vitro* recordings of detrusor contractility, animals were euthanized with overdose of sodium pentobarbital (150 mg/kg). Midline laparotomy was performed to remove the urinary bladder, which was divided in two halves longitudinally and weighed. Full-thickness strips of the bladder wall were tied to silk threads and suspended from L-shaped hooks in 15-ml organ bath chambers. The chambers were filled with the Tyrode buffer (in mM: 125 NaCl, 2.7 KCl, 23.8 NaHCO_3_, 0.5 MgCl_2_·6H_2_O, 0.4 NaH_2_PO_4_·H_2_O, 1.8 CaCl_2_, and 5.5 dextrose), maintained at 37°C, and perfused continuously with a mixture of 95% O_2_ and 5% CO_2_. After a 30-min equilibration period, the length of optimal force development (*L*_0_) was determined by increasing the length of each strip in 1.5-mm increments until maximal contractile response to electrical field stimulation (EFS; 70 V, 32 Hz, train duration of 1 ms) was achieved. The tissues were washed three times with Tyrode buffer (10 min each) to re-equilibrate the muscle. Depolarization with high-potassium chloride solution (KCl, 125 mM) was carried out next to evaluate the tonic and phasic properties of the detrusor muscle. The tissues were washed three times (10 min each) with Tyrode buffer before the application of additional drugs. One strip from each bladder was stimulated with muscarinic receptor agonist carbachol (CCh) whereas the second strip underwent stimulation with a protein kinase C (PKC) activator followed by a Rho kinase (ROK) inhibitor. Cumulative doses of CCh (10^−7^ to 10^−4^ M) were used to trigger muscle contractions and assess the contractile response to muscarinic receptor activation. Phorbol-12,13-dibiturate (PDBU, 1 μM) was used as a PKC activator followed by application of Y27632 (20 μM), a ROK inhibitor. Additional groups of mice (WT and TRPV1^−/−^) were used to evaluate the effects of ROK inhibitor on detrusor contractility induced by KCl. Contraction parameters were measured using PowerLab Lab-Chart version 7.1.2 software (ADinstruments, Colorado Springs, CO, USA). The raw traces were analyzed manually and then exported into SigmaPlot 11 Software (Systat Software, San Jose, CA, USA).

### Surgical procedure for labeling urinary bladder sensory neurons

Mice were anesthetized with 2% isoflurane and held on a warming pad inside the designated hood to minimize an investigator`s exposure to the anesthetic. A midline laparotomy was performed under the sterile conditions to gain access to the pelvic organs. The distal colon was exposed and DiI (1,1'-dioctadecyl-3,3,3'3'-tetramethylindocarbocyanine perchlorate; Molecular Probes, Eugene, OR, USA; 1.5% w/v in methanol) was injected into the colonic wall using a Hamilton syringe with 26 gauge needle at six to ten sites. The colon was placed back into the abdominal cavity and the urinary bladder was exposed for injections. Fast Blue (Polysciences Inc., Warrington, PA, USA; 1.5% w/v in water) was injected into the urinary bladder wall (detrusor) using the same approach as described for the colon. We intentionally performed double labeling to exclude cells receiving dual afferent input from the distal colon and urinary bladder as these convergent neurons would be directly affected by colonic treatments [13]. The total volume of dye injected into each organ was 20 to 25 μl. Adjacent pelvic organs were isolated with gauze to soak up any spills and prevent the labeling of adjacent structures during dye injections. Additionally, the needle was kept in place for 30 s after each injection. Any leaked dye was removed with a cotton swab before placing the organ into the pelvic cavity. Incisions were sutured in layers under the sterile conditions followed by subcutaneous injection of buprenorphine (0.5 mg/kg). Animals were allowed to recover on a warm blanket until they gained full consciousness and then were returned to their cages. Mice underwent subsequent treatments with either vehicle or TNBS 7 to 10 days after the labeling of dorsal root ganglion (DRG) neurons.

### Isolation of single sensory neurons for patch-clamp experiments

Dorsal root ganglia were dissected and removed bilaterally at L6-S2 levels. Tissues were treated with collagenase (Worthington, type 2, Biochemical Corp., Lakewood, NJ, USA) in F-12 medium (Invitrogen, Carlsbad, CA, USA) for 90 min in an incubator with 95% O_2_ and 5% CO_2_ at 37°C. Isolated ganglia were then rinsed in phosphate-buffered saline and incubated for 15 min in the presence of trypsin (Sigma-Aldrich, St. Louis, MO, USA; 1 mg/ml) at room temperature. The enzymatic reaction was terminated in Dulbecco’s modified Eagle’s medium (DMEM) containing 10% of fetal bovine serum. Single neurons were obtained by gentle trituration using fire-polished Pasteur pipettes in DMEM with trypsin inhibitor (2 mg/ml; Sigma-Aldrich, St. Louis, MO, USA) and deoxyribonuclease (DNase 1 mg/ml; Sigma-Aldrich, St. Louis, MO, USA). The cell suspension was centrifuged for 10 mins at 700 rpm (4°C), and supernatant was discarded. The pellet, containing sensory neurons, was resuspended in 2 ml of DMEM containing 10% of fetal bovine serum. Neurons were plated on poly-L-ornithine-coated 35 mm Petri dishes. Isolated cells were maintained overnight in an incubator at 37°C with 95% O_2_/5% CO_2_ and were used for electrophysiological experiments within 24 hours.

### Electrophysiological recordings of voltage gated Na^+^ currents from bladder DRG neurons

Bladder-labeled neurons were identified using specific filter for Fast Blue (UV-2A, Nikon, Tokyo, Japan) under an inverted fluorescent microscope (Ti E2000-5, Nikon). Only neurons exhibiting bright blue fluorescence (Fast Blue labeled) were used for Na^+^ current recordings using perforated whole-cell patch clamp technique. Neurons with red (colon projecting) and pink (convergent colon-bladder neurons) fluorescence were excluded from the study. For voltage clamp experiments the external solution contained (in mM): NaCl 45, TEA Chloride 30, Choline Chloride 60, KCl 5.4, MgCl_2_ 1, CaCl_2_ 1, HEPES 5, D-glucose 5.5, adjusted with NaOH to pH 7.4. Pipette solution for these experiments consisted of (in mM): L-aspartic acid 100, CsCl 30, MgCl_2_ 2, Na-ATP 5, EGTA 5, HEPES 5 adjusted with CsOH to pH 7.2. CdCl (100 μM) was added to the external solution in order to block voltage-gated calcium currents. Freshly made Amphotericin B (0.24 mg/ml, ACROS Organics, Morris Plains, NJ, USA) was added to the pipette solution for perforated whole-cell recordings. Microelectrodes were fabricated from borosilicate capillary glass (Sutter Instruments, Novato, CA, USA) and had resistances of 2 to 5 MΩ when filled with internal solution. Recordings commenced 5 min after the establishment of whole cell access. Series resistance was compensated ≥80 to 85%, and the calculated junction potential was around 5 mV. Cells were excluded from analysis if uncompensated series resistance resulted in a maximum voltage error >5 mV or if the seal or access resistance were unstable. Recordings and analysis of kinetic parameters of voltage-gated Na^+^ channels (VGSC) were performed using previously established protocols described in [16]. pCLAMP software (Axon Instruments, Union City, CA, USA) was used for data acquisition and analysis.

### Gene expression of voltage gated Na^+^ channels in lumbosacral DRG

A separate set of WT (N = 5) and TRPV1^−/−^ mice (N = 5) was used for RNA isolation and real-time PCR analysis of gene expression of VGSC including Nav1.7, Nav1.8, and Nav1.9 members. These channels were selected due to their exclusive expression in sensory ganglia and established participation in nociceptive signaling [26,27]. L6-S2 sensory ganglia were isolated bilaterally and snap-frozen in liquid nitrogen. Total RNA was extracted using Trizol reagent following the protocol from Invitrogen (Carlsbad, CA, USA) as previously described [21]. First strand cDNA was synthesized from 2 μg of the total RNA with 200 U of the superscript III reverse transcriptase (Invitrogen, number 18080–051) in the presence of 40 U RNaseOUT, 10 mM DTT, dNTP mix at 10 mM and 50 μM of Oligo (dT)_20_. Real-time PCR was run on 7500 Fast Real Time PCR system (Applied Biosystems, Foster City, CA, USA). TaqMan Gene Expression Master Mix (4369016-PEC) and TaqMan™ primer/probe kits were used for mouse Nav1.7 (Mm00450762-s1), Nav1.8 (Mm00501467-m1), and Nav1.9 (Mm00449377-m1) channels (all from Applied Biosystems, Carlsbad, CA, USA). Glyceraldehyde-3-phosphate dehydrogenase (GAPDH) gene served as an endogenous control for the quantification of gene expression. The data were analyzed using comparative Ct values as previously described [21]. For example, the Ct value for each gene of interest (Nav1.7, Nav1.8, or Nav1.9) in the control group was subtracted from that of GAPDH (housekeeping gene) to obtain the ΔCt value. The same subtraction was done in all treated groups to obtain the ΔCt. To compare the changes in the expression levels of Nav1.7, Nav1.8, and Nav1.9 genes between control and experimental tissues, these two ΔCt values were subtracted to obtain the ΔΔCt. The fold change was measured as 2^−ΔΔCt^.

### Assessment of abdominal sensitivity using von Frey filaments

Inflammation in the pelvic viscera is associated with enhanced abdominal sensitivity due to convergence of visceral and somatic inputs in the CNS. This phenomenon is known as viscerosomatic referred hyperalgesia and could be measured by using mechanical stimulation with von Frey filaments on the lower abdominal area [28]. In order to follow the dynamic changes in pelvic sensitivity of mice during the occurrence of experimental colitis, we tested animals right before the TNBS instillation (day 0 or baseline) followed by daily measurements until the peak of colonic inflammation developed (3 days post-instillation). The same approach was applied to mice in the groups with intracolonic vehicle treatments. Mice were tested in individual Plexiglas chambers (6 × 10 × 12 cm) with a stainless steel wire grid floor (acclimation period was 30 min before testing). Frequency of withdrawal responses was tested using five individual fibers with forces of 0.04, 0.16, 0.4, 1, and 4 g (Stoelting, Kiel, WI, USA). Each filament was applied for 1 to 2 s with an interstimulus interval of 5 s for a total of 10 times, and the hairs were tested in ascending order of force. All tests were performed by the same person who was unaware of the phenotype/treatment of animals. Stimulation was confined to the lower abdominal area in the general vicinity of the bladder, and care was taken to stimulate different areas within this region to avoid desensitization or ‘wind-up’ effects. Retraction of the abdomen, licking or scratching of the area of filament stimulation, and jumping-like behavior were considered as positive responses to filament stimulation as previously described [28].

### Chemicals and drugs

All chemicals were obtained from Sigma-Aldrich with the exception of DiI (Molecular Probes, Invitrogen, Eugene, OR, USA) and Fast Blue (Polysciences, Warrington, PA, USA). Isoflurane was purchased from VEDCO Inc. (St. Joseph, MO, USA) and Amphotericin B from ACROS (Organics, Morris Plains, NJ, USA). Trizol kit for RNA/protein isolation and SuperScript III Reverse Transcriptase were obtained from Invitrogen (Carlsbad, CA, USA).

### Statistical analysis

All data are expressed as the mean ± standard error of the mean (SEM). N reflects the number of animals per group and n corresponds to the number of samples in each group. Statistical significance between the groups was assessed by one-way repeated measures ANOVA followed by Bonferroni`s post hoc test when appropriate (Systat Software Inc., San Jose, CA, USA). Difference between the groups and treatments was considered statistically significant at *P* ≤0.05.

## Results

### Absence of TRPV1 receptors does not eliminate the development of colonic inflammation

**Figure 1:**
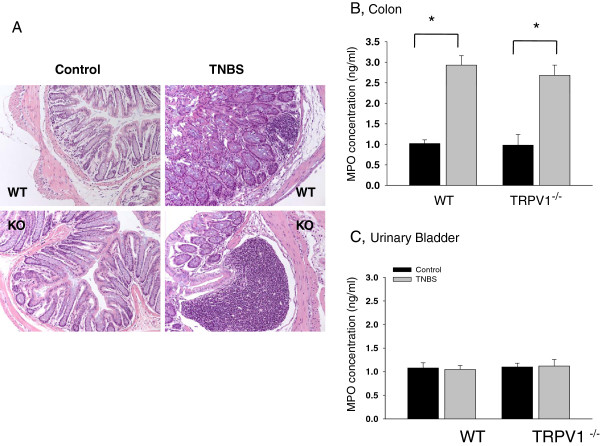
**Development of acute inflammation in the distal colon in WT and TRPV1**^**−/−**^**mice. (A)** Hematoxylin and eosin (H&E) staining of the distal colon in rats with intracolonic vehicle (Control) and 2,4,6-trinitrobenzene sulfonic acid (TNBS) treatment in wild-type (WT) and transient receptor potential vanilloid 1 knockout (TRPV1^−/−^) mice (20X). In the groups with TNBS treatment please note the signs of colonic inflammation and tissue damage including sites of hemorrhage, infiltration and disruption of the colonic crypts (c). **(B)** Concentration of myeloperoxidase (MPO) enzyme in the distal colon in mice with and without active colonic inflammation. **(C)** MPO activity in the urinary bladder is unchanged by experimental colitis. *P ≤0.05 to respective control.

Severity of inflammatory reaction induced by TNBS in WT and TRPV1^−/−^ mice was assessed by histological and biochemical methods as previously described [16,21]. Analysis of H&E-stained sections of the distal colon in both WT (N = 10) and TRPV1^−/−^ (N = 10) groups revealed that intracolonic vehicle did not evoke any detectable structural changes in the colonic wall (Figure 1A). However, TNBS treatment induced crypt segmentation, local infiltration and thickening of the muscle layer in the distal colon in both groups of animals (Figure 1A). The cytoarchitecture of the bladder wall was not affected by either treatment in all tested animals (data not shown). The MPO assay is a validated biochemical method of grading inflammation in the tissue and measures the amount of MPO enzyme released by neutrophils at the site of inflammation [29]. The concentration of the enzyme was increased 3-fold in the colon of both WT (N = 10) and TRPV1^−/−^ (N = 10) mice in TNBS groups (Figure 1B) but was unchanged in the urinary bladder (Figure 1C). Taken together, these data demonstrate substantial inflammatory changes in the distal colon during acute colitis in both WT and TRPV1^−/−^ groups suggesting that the lack of TRPV1 receptors does not prevent the colon from the development of acute inflammatory reaction.

### Urodynamic analysis of bladder function in awake unrestrained mice

**Figure 2:**
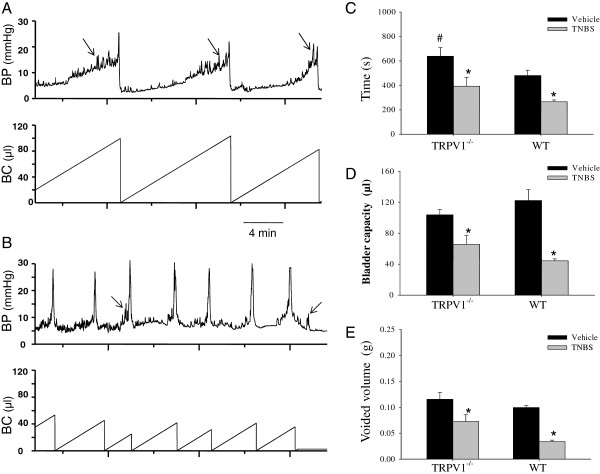
**Cystometric evaluation of bladder function before and after the development of a neurogenic bladder. (A-B)** Representative traces of the cystometrograms recorded in a freely moving transient receptor potential vanilloid 1 knockout (TRPV1^−/−^) mouse before (**A**) and after (**B**) the induction of colonic inflammation. Arrows point to non-micturition contractions. **(C)** Comparison of the duration of intermicturition interval (IMI) in wild-type (WT) and genetically modified mice. **(D)** Analysis of bladder capacity (BC) before and after 2,4,6-trinitrobenzene sulfonic acid (TNBS) treatments. **(E)** Changes in the voided volume induced by experimental colitis. BP, bladder pressure. *P ≤0.05 to respective vehicle group, #*P* ≤0.05 to WT group.

To evaluate the effects of colon-bladder cross-sensitization on the micturition parameters *in vivo*, we performed cystometric assessment of bladder function during slow continuous bladder filling in conscious mice. Cystometrograms were recorded under control conditions in WT (N = 4) and TRPV1^−/−^ (N = 5) mice followed by second evaluation at 3 to 5 days post-TNBS. Figure 2 shows cystometric traces obtained from a TRPV1^−/−^ mouse before (A) and after TNBS application (B). Urodynamic parameters were first compared to the baseline to evaluate the effects of TNBS-induced colitis on the voiding reflex in each animal followed by comparisons between the treatments and groups. Analysis of cystometrograms in WT and TRPV1^−/−^ mice at baseline showed that the average intermicturition interval was increased by 32.9% in KO animals in comparison to WT littermates (*P* ≤0.05, Figure 2C). However, non-voiding contractions were observed more frequently in TRPV1^−/−^ mice (3.1 ± 1.2 vs 1.1 ± 0.3 in WT group, *P* ≤0.05). Other urodynamic characteristics were not different between WT and KO animals.

Intracolonic TNBS had a profound effect on the function of the urinary bladder in both WT (N = 4) and TRPV1^−/−^ (N = 5) groups. At 3 to 5 days after TNBS treatment, intermicturition interval was reduced from 638 ± 70.7 s to 393.3 ± 70.8 s in KO group (*P* ≤0.05 to vehicle) and from 480.3 ± 43.1 s to 265.9 ± 14.8 s in WT group (*P* ≤0.05 to respective vehicle, Figure 2C). Likewise, bladder capacity was reduced by 64% in WT mice and by 37% in TRPV1^−/−^ littermates during colitis (*P* ≤0.05 to vehicle, Figure 2D). Occurrence of bladder dysfunction was also associated with decreased voided volume in both groups of animals (Figure 2E). In WT mice, the average voided volume per cycle was reduced by 67% during active colitis, however, in KO group, a decrease was only 36.4% (*P* ≤0.05 to vehicle, Figure 2E). Taken together, these results suggest that the effects of colitis-induced cross-sensitization on bladder function were attenuated in TRPV1^−/−^ mice.

### Differential effects of experimental colitis on the contractility of DSM in WT and TRPV1^−/−^ mice

**Figure 3:**
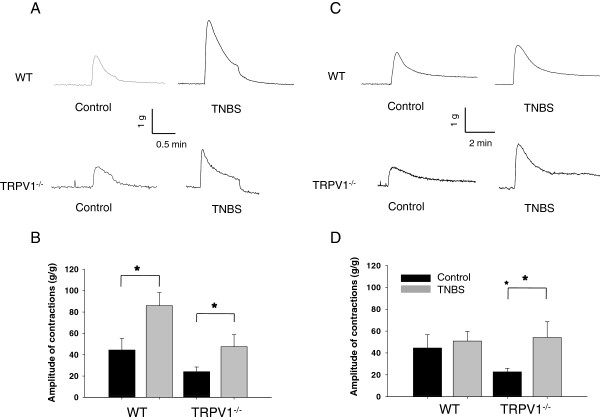
**Contractility of the detrusor muscle isolated from WT and TRPV1 KO mice. (A)** Representative raw tracers of the detrusor contractile responses to electric field stimulation (EFS) in wild-type (WT) and transient receptor potential vanilloid 1 knockout (TRPV1^−/−^) mice. **(B)** Normalized contractile response of isolated detrusor smooth muscle (DSM) strips in response to electric field stimulation (EFS, 32 Hz). **(C)** Representative raw tracers of the detrusor muscle responses to KCl stimulation in WT and TRPV1^−/−^ mice. **(D)** Changes in the peak amplitude of the detrusor muscle contractions upon stimulation with 125 mM of potassium chloride (KCl). All results are presented as mean ± SEM. Star symbol marks the statistical significance between the groups.

We performed a series of *in vitro* experiments using isolated urinary bladder strips to identify the effects of TRPV1 gene knockout on the mechanisms of detrusor smooth muscle (DSM) contractility. Isometric contractions in response to EFS at 32 Hz were evaluated in muscle strips isolated from WT and TRPV1^−/−^ mice (Figure 3A). This frequency was chosen based on our previous studies that showed significant alterations in detrusor contractility at higher frequencies during acute colonic inflammation [14,22]. The maximal amplitude of the normalized contractile response to EFS was 44.4 ± 10.8 g/g in WT group under control conditions and increased 2-fold after TNBS treatment (85.9 ± 12.3 g/g, *P* ≤0.05 to control, Figure 3B). The contractility of DSM isolated from TRPV1^−/−^ mice reached 24.1 ± 4.5 g/g in the control group, which was lower than in respective WT group (*P* = 0.11 to WT, Figure 3B). However, colonic inflammation had similar effect on the detrusor of TRPV1^−/−^ animals causing 2-fold increase in the contractile response (*P* ≤0.05 to vehicle, Figure 3B).

Stimulation of DSM strips *in vitro* with KCl (Figure 3C) allowed evaluation of the effects of TRPV1 gene knockout on DSM contractility due to depolarizing effect of KCl on smooth muscle cells [30]. The normalized amplitude of the contractile response to KCl was 22.7 ± 3.3 g/g in TRPV1^−/−^ group, which was lower than in WT group (44.5 ± 12.2 g/g), however, this difference did not reach statistical significance (*P* = 0.12 to WT, Figure 3D). Intracolonic treatment with TNBS had no effect on KCl-induced contractility of the detrusor in WT animals but caused a significant up-regulation of the response in TRPV1^−/−^ strips (*P* ≤0.05 to respective control, Figure 3D).

**Figure 4:**
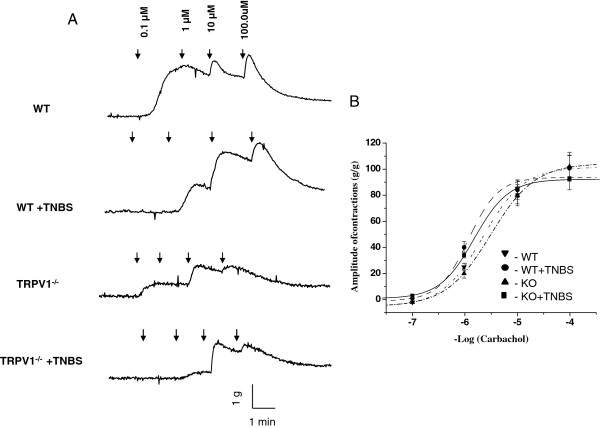
**Muscarinic receptor-dependent regulation of DSM contractions. (A)** Representative raw traces of detrusor contractions in response to 0.1 to 100 μM of carbachol (CCh) in wild-type (WT) and transient receptor potential vanilloid 1 knockout (TRPV1^−/−^) mice. **(B)** Concentration-dependent contractile responses of the detrusor muscle upon CCh stimulation.

We further investigated the effects of TRPV1 gene knockout on cholinergic regulation of DSM by studying the concentration-contractility response relationship upon application of carbachol (CCh). Cumulative addition of 10^-7^ to 10^-4^ M CCh to the bath solution resulted in concentration-dependent contractions in vehicle-treated WT animals (Figure 4A). Acute colonic inflammation diminished the response of the detrusor muscle to stimulation with CCh in both WT and TRPV1^−/−^ groups, however, the difference did not reach statistical significance (Figure 4B).

### Comparison of PKC and ROK dependent contractions in DSM upon colon-bladder cross-sensitization

**Figure 5:**
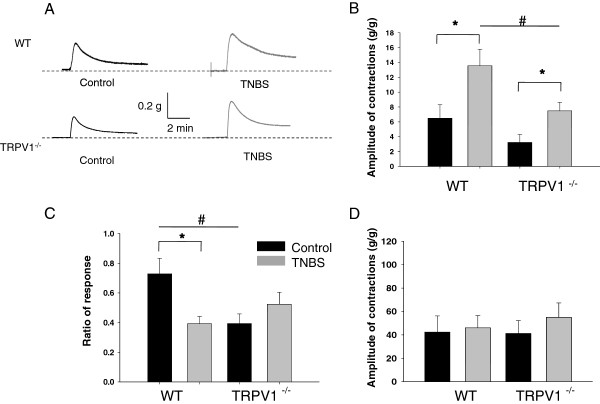
**Differential effects of colonic inflammation on PKC and ROK signaling in the bladder smooth muscle in mice lacking TRPV1 receptors. (A)** Representative raw tracers of the detrusor contractile responses to protein kinase C (PKC) activator (phorbol-12,13-dibiturate (PDBU), 1 μM) in wild-type (WT) and transient receptor potential vanilloid 1 knockout (TRPV1^−/−^) mice. **(B)** Normalized contractile response of isolated detrusor smooth muscle (DSM) strips in response to PDBU in WT and TRPV1 KO mice. **(C)** DSM relaxation response upon application of Rho-kinase (ROK) inhibitor Y27632 (20 μM) after PDBU-induced contractions. The maximal amplitude of contraction induced by PDBU was taken as 100%. Star symbols mark the statistical significance between control and treatment groups whereas pound symbols reflect significant difference between WT and KO mice. **(D)** Normalized contractile response of detrusor muscle contractions upon stimulation with high potassium chloride (KCl, 125 mM) after 30 min incubation of muscle strips with ROK inhibitor Y27632 (N = 4, n = 8 in all groups).

Increased sensitivity of DSM to KCl stimulation induced by colonic inflammation in TRPV1^−/−^ mice suggested potential modulation of the signaling changes in muscle contractile apparatus. Therefore, we tested the effects of PKC activator PDBU on detrusor contractility and Rho-kinase inhibitor Y27632 on relaxation of PDBU-induced contractile response in DSM isolated from WT and TRPV1^−/−^ mice (Figure 5A). The normalized amplitude of contractile response to PDBU was 6.5 ± 1.8 g/g in WT group (n = 6) and 3.2 ± 1.1 g/g in TRPV1^−/−^ strips (n = 8, *P* >0.05, Figure 5B). Colonic inflammation increased responses to PKC activator by 110% in WT group and by 133% in TRPV1^−/−^ group (*P* ≤0.05 to respective baseline, Figure 5B). Maximal contractile response to PDBU was taken as 100% before the application of Rho-kinase inhibitor Y27632. Application of Y27632 caused relaxation of DSM in vehicle-treated WT mice by 76%, whereas the effect on the strips from KO animals was around 40% (Figure 5C). Colonic inflammation significantly reduced the relaxation of DSM strips in WT group but had no effect in TRPV1^−/−^ mice (Figure 5C).

To further clarify the role of ROK pathway in the response of isolated detrusor to KCl stimulation, we performed an additional series of experiments using four groups of WT and TRPV1^−/−^ mice with and without colonic inflammation (N = 4, n = 8 in each group). Experimental design included initial stimulation of detrusor strips with KCl followed by wash and subsequent incubation with Rho-kinase inhibitor Y27632 for 30 min. After incubation with the drug, the contractile response of the same strips to KCl was tested again. The normalized amplitude of the initial contractile response to KCl in all tested groups was similar to that presented in Figure 3D. However, incubation with Y27632 compound did not significantly affect the amplitude of KCl-induced contractions in either group (Figure 5D) suggesting that the ROK-dependent component of the detrusor response to KCl was not directly affected by the knockout of TRPV1 gene.

### Colonic inflammation up-regulates voltage-gated sodium channels in bladder sensory neurons from WT but not TRPV1^−/−^ mice

In the next set of experiments we aimed to determine if knockdown of TRPV1 gene would affect excitability of bladder sensory neurons via effects on voltage-gated sodium channels (VGSC) as we previously reported for a rat model of cross-sensitization [16,31]. Retrograde labeling of lumbosacral sensory neurons revealed three populations of isolated DRG neurons: colon-projecting, bladder-projecting and colon-bladder convergent neurons as previously detected [13,15]. We intentionally performed double labeling to exclude from the experiments neurons receiving dual afferent input from the distal colon and urinary bladder as these neurons would be directly affected by colonic treatments. The number of double labeled cells was around 10% as previously reported [13,15]. Only neurons labeled with Fast Blue (bladder projecting) underwent electrophysiological evaluation and were used for data analysis.

**Figure 6:**
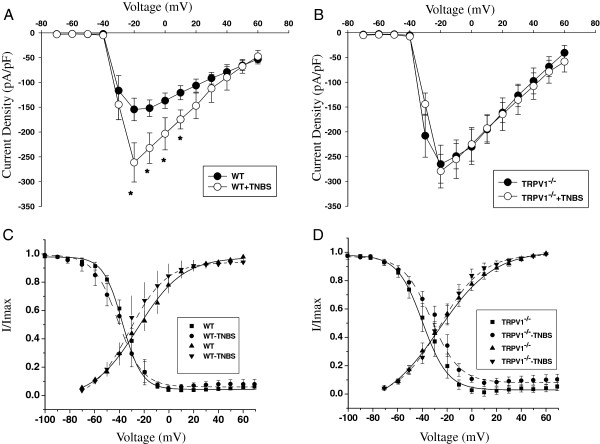
**Effects of TRPV1 gene knockout on function of voltage-gated Na**^**+**^**currents recorded in bladder sensory neurons. (A)** Current–voltage (I-V) relationship of total Na^+^ current recorded in bladder dorsal root ganglia (DRG) neurons after intracolonic 2,4,6-trinitrobenzene sulfonic acid (TNBS) treatment in wild-type (WT) mice. *P ≤0.05 when compared to vehicle group. **(B)** Current–voltage (I-V) relationship of total Na^+^ current recorded in bladder sensory neurons isolated from transient receptor potential vanilloid 1 knockout (TRPV^−/−^) animals. **(C)** Voltage dependence of steady-state activation and inactivation of total Na^+^ current recorded in bladder DRG neurons from WT mice. The steady-state activation of voltage-gated sodium channels (VGSC) was assessed by a three-pulse protocol with a negative pre-pulse to −110 mV and a series of short pulses of 10 ms duration from −110 mV to +70 mV to activate Na+ currents. The amplitude of steady-state inactivation was measured at 0 mV after 150 ms depolarizing pulses ranging from −100 mV to 70 mV. **(D)** Activation and inactivation kinetics of total Na^+^ current recorded in lumbosacral bladder neurons isolated from TRPV1 KO mice.

The current–voltage (I-V) relationship of total Na^+^ current recorded upon membrane depolarization in bladder afferent neurons from WT mice is shown in Figure 6A. Acute colonic inflammation increased the peak amplitude of total Na^+^ current by 2-fold from −153.4 ± 17.4 pA/pF at −20 mV in the control group (N = 6, n = 9) to −256.5 ± 24.1 pA/pF in the acute colitis group (N = 5, n = 7, *P* ≤0.05, Figure 6A). The amplitude of total Na^+^ current recorded in bladder DRG neurons from TRPV1^−/−^ mice (N = 6, n = 8) with vehicle treatment was enlarged in comparison to respective WT group (N = 5, n = 7), however, intracolonic TNBS had no effect on Na^+^ current in KO animals (Figure 6B).

We next assessed the kinetic parameters of Na^+^ currents after the induction of experimental colitis. The steady-state activation was studied by using a three-pulse protocol with a negative pre-pulse to −110 mV and a series of short pulses (10 ms duration) to activate Na^+^ currents as previously described [16]. The amplitude of steady-state activation was measured at the peak of tail current upon the voltage step to −70 mV, normalized and plotted as I/I_max_ against the voltage. The amplitude of steady-state inactivation was measured at a series of membrane depolarizing steps ranging from −100 mV to +70 mV. In the group of WT mice, neither rate of activation, nor steady-state inactivation kinetics of total Na^+^ current in bladder sensory neurons were affected by acute colitis (Figure 6C). However, in TRPV1^−/−^ neurons, acute colitis induced a rightward shift in steady-state inactivation by 7 mV (V_1/2_ = −39.7 ± 1.9 mV in the vehicle group vs −32.7 ± 1.7 mV in TNBS group, *P* ≤0.05, Figure 6D).

### Up-regulation of gene expression of VGSC in lumbosacral DRG by colitis is attenuated in TRPV1 KO mice

**Figure 7:**
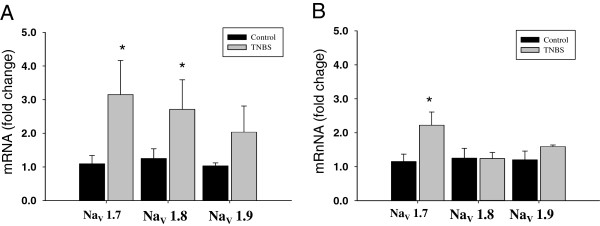
**Gene expression of voltage-gated sodium channels Nav1.7, Nav1.8 and Nav1.9 in L6-S2 dorsal root ganglia. (A)** Bar chart shows the mRNA level changes of voltage-gated sodium channels (VGSC) in lumbosacral dorsal root ganglia (DRG) of wild-type mice with and without neurogenic bladder dysfunction induced by colonic inflammation. **(B)** mRNA levels for Nav1.7, Nav1.8 and Nav1.9 in L6-S2 DRG isolated from transient receptor potential vanilloid 1 knockout (TRPV1^−/−^) mice. A star symbol reflects statistical significance between control and treatment groups (*P* ≤0.05).

To correlate the observed functional changes of the recorded VGSC in TRPV1^−/−^ mice with potential effects on gene expression of VGSC in sensory neurons involved in nociceptive signaling, we performed quantitative RT-PCR analysis. This set of experiments was carried out on the whole L6-S2 ganglia due to the presence of a limited number of retrogradely labeled bladder neurons. The DRG from both vehicle and TNBS groups were harvested 3 days post-treatment. The comparison of mRNA levels of VGSC between WT (N = 4) and TRPV1^−/−^ (N = 4) mice did not reveal significant differences under control physiological conditions (data not shown). mRNA expression of VGSC in L6-S2 DRG isolated from WT mice showed 3-fold up-regulation for Nav1.7 channel and 2-fold increase for Nav1.8 channel by experimental colitis (N = 5, *P* ≤0.05 to respective vehicle, Figure 7A). The Nav1.9 mRNA level was also increased in WT group, but did not reach the level of statistical significance. The effects of colonic inflammation on gene expression of Nav1.7, Nav1.8 and Nav1.9 channels were diminished in TRPV1^−/−^ ganglia showing 2-fold up-regulation for Nav1.7 channel only (N = 5, *P* ≤0.05 to vehicle, Figure 7B).

### Lack of TRPV1 receptors delays the development of abdominal hypersensitivity during colon-bladder cross-talk

**Figure 8:**
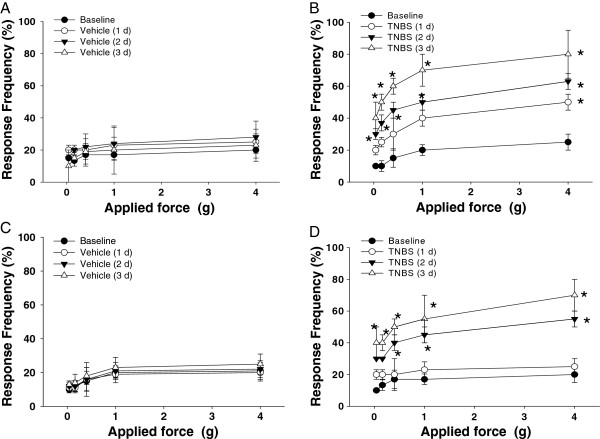
**Differences in visceral sensitivity between WT and TRPV1 KO mice in response to mechanical stimulation of the lower abdominal region. (A)** Abdominal responses to Von Frey filaments in wild-type (WT) mice with vehicle instillations (N = 4). **(B)** Responses to pelvic stimulation in WT mice before and after intracolonic treatment with 2,4,6-trinitrobenzene sulfonic acid (TNBS) (N = 5). **(C)** Time course of the responses to mechanical stimulation of the lower pelvic area in transient receptor potential vanilloid 1 knockout (TRPV1 KO) mice before and after intracolonic instillations of the vehicle (N = 4). **(D)** Summary of the responses to pelvic stimulation recorded in TRPV^−/−^ male mice during experimental colitis (N = 6). Presented data are mean percentages of response frequency (± SE) before (baseline) and at days 1, 2, and 3 post-treatment. ANOVA indicated a significant increase in response frequency in WT mice within 3 days post-TNBS treatment, whereas in KO mice the difference reached statistical significance only at days 2 and 3 after the induction of colonic inflammation.

Abdominal sensitivity was daily tested in WT and TRPV1^−/−^ mice at the baseline and after intracolonic treatments with either vehicle or TNBS. Figure 8 summarizes the frequency of responses in the lower abdominal area in all tested mice. Mechanical stimulation of the pelvic area under control conditions (vehicle treatment) in both WT (N = 4, Figure 8A) and TRPV1^−/−^ mice (N = 4, Figure 8C) resulted in a response frequency that correlated with the applied force, reaching around 20% at the maximal tested force of 4 g during the entire tested period . In the group with TNBS treatment, WT mice showed increased sensitivity to the filament testing and responses reached 40% at applied forces of 1 and 4 g (Figure 8B, N = 5, *P* ≤0.05 to baseline). An increase in pelvic sensitivity became most significant at 3 days after the treatment with statistically higher percentage of responses at all tested forces (Figure 8B, N = 6, *P* ≤0.05 to baseline). Mice in TRPV1^−/−^ group with had similar baseline sensitivity to von Frey filaments when compared to WT group. TNBS application did not evoke significant changes in withdrawal response 1 day after the treatment but increased abdominal sensitivity at days 2 and 3 up to 40% and 65%, respectively, for 4 g filaments (*P* ≤0.05 to vehicle, Figure 8D). These results provide evidence that colonic inflammation leads to an increased viscerosomatic response associated with abdominal hyperalgesia and discomfort, and the lack of TRPV1 receptors delays the development of viscerosomatic hyperalgesia in KO mice.

## Discussion

This is the first report which evaluated viscero-visceral cross-reflexes in TRPV1 KO mice and confirmed previously reported data that transient inflammation of the distal colon triggers the development of neurogenic bladder dysfunction via TRPV1-related pathways [14,16,18,19,21,32]. Our results provide direct evidence that the lack of TRPV1 receptors does not prevent the occurrence of colon-bladder cross-sensitization induced by transient colitis, however, several important physiological characteristics of bladder function are altered by the knockout of TRPV1 gene. The major differences included prolonged intermicturition interval in KO animals followed by reduced urodynamic responses during active colitis; up-regulation of DSM contractility in response to KCl in TRPV1^−/−^ mice with inflamed colon; diminished relaxation of DSM in transgenic animals in the presence of ROK inhibitor; attenuated effects of colonic inflammation on expression and function of VGSC in bladder sensory neurons from TRPV1^−/−^ mice; and delayed development of abdominal hypersensitivity upon colon-bladder cross-talk in genetically modified animals.

An increasing number of clinical reports support animal studies that peripheral and central cross-sensitization may underlie unknown etiology of functional gastrointestinal and urologic disorders associated with abdominal discomfort, visceral hypersensitivity, and chronic pelvic pain [3,4,33]. While it is understood that animal models cannot fully mimic human co-morbid conditions, they provide valuable insight to study the underlying mechanisms. In the present study, we established that the absence of TRPV1 did not affect the severity of TNBS-induced colitis in transgenic mice. Several previous reports presented contradictory results on the role of TRPV1 in inflammation suggesting that the impact of TRPV1 involvement is model-, dose-, and species-dependent [34]. For instance, Okayama *et al.*[35] established that desensitization of TRPV1 receptors with potent agonists elevated histological changes in the colon in dextran sulfate sodium (DSS) model of colitis, whereas other groups demonstrated a diminished level of the developed inflammatory reaction in the distal colon of rats [21,36-38]. Experiments on TRPV1 KO mice using a single dose of dinitrobenzene sulfonic acid reported an increased susceptibility of the colon to the applied inflammatory agent [39], however, the effects of DDS on TRPV1 KO mice were clearly dose-dependent. In 2% DSS-treated group, the lack of TRPV1 receptors decreased the severity of the induced colitis, however, this difference was not observed for 5% DSS, when much severe inflammatory reaction developed [40]. While the severity of inflammatory response could be modulated by activation of TRPV1 receptors, it is also possible that nerve fibers sensitive to capsaicin can be activated by non-TRPV1-dependent pathways during pathological conditions [40]. In a model of cutaneous inflammation, the lack of TRPV1 receptors did not alter the leukocyte accumulation suggesting participation of both neurogenic and non-neurogenic mechanisms [41]. Importantly, the activation of sensory nerves was still observed in TRPV1 KO mice providing evidence that neurogenic inflammation may also develop via TRPV1 independent pathways [41].

Our analysis of urodynamic parameters in awake mice confirmed previously reported involvement of TRPV1 in regulation of urinary bladder function under both normal and pathophysiological conditions. The intermicturition interval in TRPV1^−/−^ mice was increased along with the number of non-voiding contractions at the baseline, and the effects of experimental colitis on bladder capacity and voided volume were diminished in comparison to WT littermates. Our results are in line with the previous studies which reported increased bladder capacity and non-micturition contractions [42] as well as attenuated bladder distension-induced afferent discharge in TRPV1^−/−^ mice [43]. Participation of TRPV1 in bladder sensitivity and pain was also demonstrated in several animal models of direct bladder inflammation [44-46]. These combined results suggest that TRPV1 pathways are involved in inflammatory and neurogenic bladder dysfunctions, and also contribute to the development of pelvic organ cross-sensitization.

Previous studies from our [14,16,20] and other [12,15,17] laboratories established that colon-bladder cross-sensitization develops predominantly, but not exclusively, via neural pathways. It is well established that TRPV1 channels are expressed not only on sensory neurons but also on peripheral afferents supplying visceral organs of the gastrointestinal and genitourinary tracts [24,25,47]. Our data from isolated DSM strips clearly demonstrate that the absence of TRPV1 expression on bladder afferents did not cause significant changes in contractile responses of the detrusor upon normal physiological conditions. However, some modulatory changes in the signaling cascades became more evident during additional stimulation like active colonic inflammation or PKC activation. Specifically, we found that the contractile response of DSM to KCl stimulation was enhanced by experimental colitis in KO animals. KCl is used as a tool to bypass G protein-coupled receptor stimulation and activate smooth muscle by changing potassium equilibrium potential and triggering membrane depolarization (reviewed in [30]). Activation of TRPV1, a cation channel permeable mostly to Ca^2+^, has the same depolarizing effect on the cell but the major mechanism includes massive influx of Ca^2+^ through the pore of the channel [48]. Recent studies established that activation of TRPV1 by capsaicin, a potent TRPV1 agonist, is associated with PKC activation [49,50], and the opening of TRPV1 channels is promoted by channel phosphorylation [51]. PKC modulation of TRPV1 channel function was previously shown to occur under conditions of chronic pain resulting from nerve damage or inflammation [50].

Rho kinase (ROK) pathway in bladder detrusor smooth muscle was shown to be involved in the contractile response of the isolated DSM to KCl [30]. ROK pathway is critical for the maintenance of basal tone in DSM and serves as a common final pathway of various contractile stimuli in many species [52]. ROK directly phosphorylates and inactivates myosin light chain (MLC) phosphatase, ultimately increasing the phosphorylation state of myosin and facilitating contraction [53]. Y27632, a ROK inhibitor, was shown to reduce KCl-induced contractile force without inhibition of KCl-induced increases in intracellular Ca^2+^[54-57], but with concomitant inhibition of MLC phosphorylation [55,56].

The relationship between TRPV1 and ROK in the detrusor muscle is currently not established, but activation of TRPV1 resulted in inhibition of ROK in vascular smooth muscle [58]. Fujimoto *et al.* previously investigated the effects of capsaicin-induced relaxation on KCl-induced contractions and phosphorylation of MLC in ileal smooth muscle of rats [59]. They established that capsaicin relaxed intestinal smooth muscle after KCl-induced contraction and this effect was accompanied by a decrease in MLC_20_ phosphorylation [59]. To clarify the effects of ROK inhibition on KCl-induced contractility of the DSM in our setting, we performed a series of experiments where DSM was tested with KCl after 30-min incubation with a ROK inhibitor. Our results suggest that without preliminary stimulation with PKC activator PDBU (as we did in the primary set of experiments), the effects of ROK inhibition on the contractility of the detrusor induced by KCl were insignificant between the WT and TRPV1^−/−^ groups (Figure 5D). This data confirms the absence of the direct effect of TRPV1 knockdown on detrusor muscle contractile apparatus. However, the modulatory effects of PKC and ROK pathways could be associated with indirect mechanisms such as the altered release of sensory neuropeptides from TRPV1 fibers triggered by colonic inflammation. TRPV1-expressing afferent nerves are well known for their ‘efferent’ function associated with the release of neuropeptides, mainly tachykinins and calcitonin gene-related peptide (CGRP) upon peripheral stimulation [60]. It is possible that the absence of TRPV1 expression on afferent nerves in TRPV1^−/−^ mice can modulate tachykinin levels in the detrusor which, in turn, are capable of suppressing endogenous proteases like ROK [61]. Further studies are warranted to evaluate the relationship between detrusor contractility, expression of TRPV1 channels on bladder afferents and release of sensory neuropeptides in colon-bladder cross-talk.

Sensory neurons receiving afferent input from the pelvic viscera play a key role in the development of peripheral cross-sensitization [13,15]. They express several types of ion channels including TRPV1 and VGSC, which are well-known transducers of nociceptive processing in pain pathways [26,27,48]. Excitability of sensory afferents is defined by expression and function of VGSC [26,27]. Single cell analysis of Na^+^ channel transcripts indicated that sensory neurons innervating the pelvic viscera express a combination of tetrodotoxin-sensitive and -resistant Na^+^ channels, which consist mostly of Na_v_1.6, Na_v_1.7, Na_v_1.8 and Na_v_1.9 members [62]. In this study, the peak amplitude of total sodium current in neurons from vehicle-treated TRPV1^−/−^ mice was greater than that of neurons from the vehicle-treated WT mice. Multiple sodium channel isoforms are expressed in DRG neurons and play a central role in neuronal electrogenesis. However, a number of other ion channels also confer electrical excitability of neurons including voltage-gated calcium and potassium channels. Interactions between different ion channels and co-expressed molecules lead to the occurrence of multiple ionic conductances, which shape the cells' firing patterns, and confirm that regulation of neuronal excitability is a complex process. Under non-pathological conditions, the firing properties of DRG neurons are usually maintained within a circumscribed range. This is a result of homeostatic regulation of ion channel expression, post-translational modification, and/or interaction with binding partners or modulators [63]. While variations in the level of expression of any VGSC isoform present within DRG neurons could affect their level of excitability, computational models have demonstrated that multiple, distinct sets of membrane parameters can produce similar levels of activity. For instance, it has been proposed that changes in expression of channel ‘B' can compensate for changes in expression of channel ‘A' to maintain excitability within a particular range [64]. Indeed, it was demonstrated that overexpression of Shal potassium channel gene in somatogastric ganglion neurons caused a large increase in potassium current but little change in the neuron's firing properties due to a compensatory increase in expression of a hyperpolarization-activated inward current [65]. Likewise, in Purkinje neurons isolated from Na_V_1.6^−/−^ mice, sodium current density was reduced, however, an up-regulation of calcium channels maintained excitability near its normal level [66]. Therefore, while we observed enhanced amplitude of VGSC current in TRPV1^−/−^ neurons, we could not confirm that these neurons were hyperexcitable in comparison to WT phenotype. Additional extensive studies are warranted to perform comprehensive evaluation of neuronal excitability in genetically modified animals.

Studies utilizing animal models of acute and chronic inflammation in different parts of gastrointestinal [11,67,68] and genitourinary [44,69] systems showed an increased excitability of DRG neurons receiving direct input from the inflamed organs. Previously published data from our group also established that colonic inflammation increased excitability of bladder DRG neurons via an up-regulation of VGSC [16]. However, cross-channel interactions between TRPV1 and VGSC at the membrane level have not been fully investigated. Under *in vitro* conditions, application of capsaicin usually leads to a significant block of VGSC [70], but intraluminal application of TRPV1 agonists *in vivo* causes an up-regulation of VGSC on the cell soma and is associated with increased excitability of sensory neurons [16]. Additional *in vitro* studies revealed that capsaicin-induced blockade of VGSC is concentration-dependent [71]. It was also established that the block of VGSC by low concentrations of capsaicin was reversed in TRPV1^−/−^ mice [71]. There are a few suggested mechanisms underlying functional interconnection between TRPV1 and VGSCs. Indirect pathways include intercrossing of TRPV1 and VGSC signaling pathways via modulation of the same transcriptional/translational factors or recruitment of second messengers (for example cAMP or intracellular Ca^2+^). Direct pathways involve TRPV1-VGSC interactions within a complex as it was proven for other channels [72-74]. It is possible that genetic knockdown of TRPV1 gene in sensory neurons could lead to compensatory changes by up- or down-regulation of other ion channels. Since VGSCs are the main source of positive charge influx into sensory neurons, they could have been affected by TRPV1 gene knockout during development. The future research steps should determine if one of the VGSCs expressed in DRG has some sort of capsaicin receptor and whether modulatory subunits of both channels are involved in TRPV1-VGSC ‘cross-talk’.

Inflammation in the pelvic viscera is associated with enhanced abdominal sensitivity due to convergence of visceral and somatic inputs in the nervous system. This phenomenon is known as viscerosomatic referred hyperalgesia and could be measured by using mechanical stimulation with von Frey filaments on the lower abdominal area [28]. The referred hyperalgesia is proportional to the intensity of the spontaneous visceral pain-related behavior expressed by the animals. Increased responses of the lower abdominal wall to mechanical stimulation are reflective of and correlate with pain and discomfort arising from inflamed pelvic organs [75]. Our behavioral results established that the development of abdominal hypersensitivity induced by colitis upon mechanical stimulation of the lower pelvic region was delayed in TRPV1^−/−^ mice. This observation is in line with the previously published studies confirming participation of TRPV1 in the development of abdominal hyperalgesia and inflammatory pain [76]. Wang *et al*. determined that the lack of functional TRPV1 did not eliminate histological evidence of bladder inflammation in either cyclophosphamide- or acrolein-induced models of bladder inflammation [46]. This group also established that abdominal hyper-reactivity and cutaneous allodynia were abolished in TRPV1^−/−^ mice. Likewise, selective TRPV1 antagonists attenuated mechanical allodynia and hyperalgesia in rat models of neuropathic pain [76-78]. However, TRPV1^−/−^ mice did not reveal a significantly altered phenotype in the partial sciatic nerve ligation model of neuropathic pain [79]. The use of RNA interference technologies further confirmed model- and species-associated differences in test results from genetically and functionally modified animals. Functional knockdown of TRPV1 using shRNA showed diminished behavioral responses to intraplantar injection of capsaicin, enhanced paw withdrawal latencies to heat, and diminished tactile hypersensitivity in an injury-induced neuropathic pain model [80,81]. siRNA treatment also diminished spontaneous visceral pain behavior induced by capsaicin application to the rectum of mice [77]. Interestingly, spinal nerve injured TRPV1 knockout but not shRNA-treated animals developed mechanical allodynia and hypersensitivity [80]. These results indicate that neither the complete loss nor a profound reduction in TRPV1 channels can completely eliminate the effects of visceral or cutaneous noxious stimulation on behavioral responses suggesting that TRPV1 is not the only player in nociceptive signaling.

## Conclusions

In summary, the results of our extensive study clarified the role of TRPV1 in the development of colon-bladder cross-sensitization and associated dysfunction in the lower urinary tract. Knockout of TRPV1 gene changed several important physiological characteristics of bladder function including prolonged intermicturition interval along with reduced urodynamic responses during active colitis in genetically modified mice; up-regulation of DSM contractility in response to KCl in TRPV1^−/−^ mice with inflamed colon; diminished relaxation of DSM in transgenic animals in the presence of ROK inhibitor, and attenuated effects of colonic inflammation on VGSC in bladder sensory neurons. The mechanisms underlying neurogenic bladder dysfunctions due to pelvic organ cross-interactions require further studies to provide valuable information for the development of new pharmacological approaches and therapeutic strategies for the treatment of co-morbid conditions characterized by functional pelvic pain arising from gastrointestinal or genitourinary systems.

## Abbreviations

CCh: carbachol; CNS: central nervous system; CPP: chronic pelvic pain; DAI: disease activity index; DMEM: Dulbecco’s modified Eagle’s medium; DRG: dorsal root ganglia; DSM: detrusor smooth muscle; DSS: dextran sulfate sodium; EFS: electric field stimulation; ELISA: enzyme-linked immunosorbent assay; GI: gastrointestinal; IBS: irritable bowel syndrome; IC/BPS: interstitial cystitis/bladder pain syndrome; KC1: potassium chloride; KO: knockout; MLC: myosin light chain; MPO: myeloperoxidase; PDBU: phorbol-12,13-dibiturate; PKC: protein kinase C; ROK: Rho kinase; PCR: polymerase chain reaction; TNBS: 2,4,6-trinitrobenzene sulfonic acid; TRPV1: transient receptor potential vanilloid 1; TRPV1^−/−^: TRPV1 knockout; VGSC: voltage-gated sodium channels; WT: wild type.

## Competing interests

The authors declare that they have no competing interests.

## Authors’ contributions

APM and QL designed the study; QL, X-QP, ANV, TSA, SC and SAZ performed the experiments; QL, ANV, TSA, X-QP, and APM analyzed the data; QL X-QP, ANV and APM prepared the figures; QL and APM edited and finalized the manuscript. All authors approved the final version of the paper.

## References

[B1] Alagiri M Chottiner S Ratner V Slade D Hanno PM Interstitial cystitis: unexplained associations with other chronic disease and pain syndromes Urology 1997 49 52 57 10.1016/S0090-4295(99)80332-X 9146002

[B2] Francis CY Duffy JN Whorwell PJ Morris J High prevalence of irritable bowel syndrome in patients attending urological outpatient departments Dig Dis Sci 1997 42 404 407 10.1023/A:1018838507545 9052526

[B3] Rodriguez MA Afari N Buchwald DS Evidence for overlap between urological and nonurological unexplained clinical conditions J Urol 2009 182 2123 2131 10.1016/j.juro.2009.07.036 19758633 PMC2957306

[B4] Cory L Harvie HS Northington G Malykhina A Whitmore K Arya L Association of neuropathic pain with bladder, bowel and catastrophizing symptoms in women with bladder pain syndrome J Urol 2012 187 503 507 10.1016/j.juro.2011.10.036 22177143 PMC4465103

[B5] Terruzzi V Magatti F Quadri G Tenore C Minoli G Belloni C Bladder dysfunction and irritable bowel syndrome Am J Gastroenterol 1992 87 1231 1232 1519598

[B6] Whorwell PJ Lupton EW Erduran D Wilson K Bladder smooth muscle dysfunction in patients with irritable bowel syndrome Gut 1986 27 1014 1017 10.1136/gut.27.9.1014 3758813 PMC1433792

[B7] Giamberardino MA Costantini R Affaitati G Fabrizio A Lapenna D Tafuri E Mezzetti A Viscero-visceral hyperalgesia: characterization in different clinical models Pain 2010 151 307 322 10.1016/j.pain.2010.06.023 20638177

[B8] Latremoliere A Woolf CJ Central sensitization: a generator of pain hypersensitivity by central neural plasticity J Pain 2009 10 895 926 10.1016/j.jpain.2009.06.012 19712899 PMC2750819

[B9] Malykhina AP Neural mechanisms of pelvic organ cross-sensitization Neuroscience 2007 149 660 672 10.1016/j.neuroscience.2007.07.053 17920206

[B10] Brumovsky PR Gebhart GF Visceral organ cross-sensitization - an integrated perspective Auton Neurosci 2010 153 106 115 10.1016/j.autneu.2009.07.006 19679518 PMC2818077

[B11] Bielefeldt K Lamb K Gebhart GF Convergence of sensory pathways in the development of somatic and visceral hypersensitivity Am J Physiol Gastrointest Liver Physiol 2006 291 G658 G665 10.1152/ajpgi.00585.2005 16500917

[B12] Brumovsky PR Feng B Xu L McCarthy CJ Gebhart GF Cystitis increases colorectal afferent sensitivity in the mouse Am J Physiol Gastrointest Liver Physiol 2009 297 G1250 G1258 10.1152/ajpgi.00329.2009 19779012 PMC2850082

[B13] Malykhina AP Qin C Greenwood-van MB Foreman RD Lupu F Akbarali HI Hyperexcitability of convergent colon and bladder dorsal root ganglion neurons after colonic inflammation: mechanism for pelvic organ cross-talk Neurogastroenterol Motil 2006 18 936 948 10.1111/j.1365-2982.2006.00807.x 16961697

[B14] Asfaw TS Hypolite J Northington GM Arya LA Wein AJ Malykhina AP Acute colonic inflammation triggers detrusor instability via activation of TRPV1 receptors in a rat model of pelvic organ cross-sensitization Am J Physiol Regul Integr Comp Physiol 2011 300 R1392 R1400 10.1152/ajpregu.00804.2010 21474425 PMC3119151

[B15] Christianson JA Liang R Ustinova EE Davis BM Fraser MO Pezzone MA Convergence of bladder and colon sensory innervation occurs at the primary afferent level Pain 2007 128 235 243 10.1016/j.pain.2006.09.023 17070995 PMC1892845

[B16] Lei Q Malykhina AP Colonic inflammation up-regulates voltage-gated sodium channels in bladder sensory neurons via activation of peripheral transient potential vanilloid 1 receptors Neurogastroenterol Motil 2012 24 575 e257 10.1111/j.1365-2982.2012.01910.x 22420642 PMC3352963

[B17] Pezzone MA Liang R Fraser MO A model of neural cross-talk and irritation in the pelvis: implications for the overlap of chronic pelvic pain disorders Gastroenterology 2005 128 1953 1964 10.1053/j.gastro.2005.03.008 15940629

[B18] Ustinova EE Fraser MO Pezzone MA Colonic irritation in the rat sensitizes urinary bladder afferents to mechanical and chemical stimuli: an afferent origin of pelvic organ cross-sensitization Am J Physiol Renal Physiol 2006 290 F1478 F1487 10.1152/ajprenal.00395.2005 16403832

[B19] Xia CM Gulick MA Yu SJ Grider JR Murthy KS Kuemmerle JF Akbarali HI Qiao LY Up-regulation of brain-derived neurotrophic factor in primary afferent pathway regulates colon-to-bladder cross-sensitization in rat J Neuroinflammation 2012 9 30 10.1186/1742-2094-9-30 22335898 PMC3298724

[B20] Qin C Malykhina AP Akbarali HI Foreman RD Cross-organ sensitization of lumbosacral spinal neurons receiving urinary bladder input in rats with inflamed colon Gastroenterology 2005 129 1967 1978 10.1053/j.gastro.2005.09.013 16344065

[B21] Pan XQ Gonzalez JA Chang S Chacko S Wein AJ Malykhina AP Experimental colitis triggers the release of substance P and calcitonin gene-related peptide in the urinary bladder via TRPV1 signaling pathways Exp Neurol 2010 225 262 273 10.1016/j.expneurol.2010.05.012 20501335 PMC2939259

[B22] Noronha R Akbarali H Malykhina A Foreman RD Greenwood-van MB Changes in urinary bladder smooth muscle function in response to colonic inflammation Am J Physiol Renal Physiol 2007 293 F1461 F1467 10.1152/ajprenal.00311.2007 17715261

[B23] Caterina MJ Schumacher MA Tominaga M Rosen TA Levine JD Julius D The capsaicin receptor: a heat-activated ion channel in the pain pathway Nature 1997 389 816 824 10.1038/39807 9349813

[B24] Avelino A Cruz F TRPV1 (vanilloid receptor) in the urinary tract: expression, function and clinical applications Naunyn Schmiedebergs Arch Pharmacol 2006 373 287 299 10.1007/s00210-006-0073-2 16721555

[B25] Birder LA Birder LA Kanai AJ de Groat WC Kiss S Nealen ML Burke NE Dineley KE Watkins S Reynolds IJ Caterina MJ Vanilloid receptor expression suggests a sensory role for urinary bladder epithelial cells Proc Natl Acad Sci USA 2001 98 13396 13401 10.1073/pnas.231243698 11606761 PMC60882

[B26] Dib-Hajj SD Cummins TR Black JA Waxman SG Sodium channels in normal and pathological pain Annu Rev Neurosci 2010 33 325 347 10.1146/annurev-neuro-060909-153234 20367448

[B27] Waxman SG Channel, neuronal and clinical function in sodium channelopathies: from genotype to phenotype Nat Neurosci 2007 10 405 409 10.1038/nn1857 17387329

[B28] Rudick CN Chen MC Mongiu AK Klumpp DJ Organ cross talk modulates pelvic pain Am J Physiol Regul Integr Comp Physiol 2007 293 R1191 R1198 10.1152/ajpregu.00411.2007 17626130

[B29] Krawisz JE Sharon P Stenson WF Quantitative assay for acute intestinal inflammation based on myeloperoxidase activity. Assessment of inflammation in rat and hamster models Gastroenterology 1984 87 1344 1350 6092199

[B30] Ratz PH Berg KM Urban NH Miner AS Regulation of smooth muscle calcium sensitivity: KCl as a calcium-sensitizing stimulus Am J Physiol Cell Physiol 2005 288 C769 C783 15761211 10.1152/ajpcell.00529.2004

[B31] Malykhina AP Qin C Foreman RD Akbarali HI Colonic inflammation increases Na+ currents in bladder sensory neurons Neuroreport 2004 15 2601 2605 10.1097/00001756-200412030-00008 15570160

[B32] Ravnefjord A Brusberg M Kang D Bauer U Larsson H Lindstrom E Martinez V Involvement of the transient receptor potential vanilloid 1 (TRPV1) in the development of acute visceral hyperalgesia during colorectal distension in rats Eur J Pharmacol 2009 611 85 91 10.1016/j.ejphar.2009.03.058 19344705

[B33] Chelimsky G Safder S Chelimsky T FGIDs in children are associated with many nonpsychiatric comorbidities: the tip of an iceberg? J Pediatr Gastroenterol Nutr 2012 54 690 691 10.1097/MPG.0b013e3182496b1f 22241512

[B34] Storr M TRPV1 in colitis: is it a good or a bad receptor? - a viewpoint Neurogastroenterol Motil 2007 19 625 629 10.1111/j.1365-2982.2007.00946.x 17539893

[B35] Okayama M Tsubouchi R Kato S Takeuchi K Protective effect of lafutidine, a novel histamine H2-receptor antagonist, on dextran sulfate sodium-induced colonic inflammation through capsaicin-sensitive afferent neurons in rats Dig Dis Sci 2004 49 1696 1704 15573930 10.1023/b:ddas.0000043389.96490.76

[B36] Goso C Evangelista S Tramontana M Manzini S Blumberg PM Szallasi A Topical capsaicin administration protects against trinitrobenzene sulfonic acid-induced colitis in the rat Eur J Pharmacol 1993 249 185 190 10.1016/0014-2999(93)90431-G 8287899

[B37] Kihara N de la Fuente SG Fujino K Takahashi T Pappas TN Mantyh CR Vanilloid receptor-1 containing primary sensory neurones mediate dextran sulphate sodium induced colitis in rats Gut 2003 52 713 719 10.1136/gut.52.5.713 12692058 PMC1773638

[B38] Kimball ES Wallace NH Schneider CR D'Andrea MR Hornby PJ Vanilloid receptor 1 antagonists attenuate disease severity in dextran sulphate sodium-induced colitis in mice Neurogastroenterol Motil 2004 16 811 818 10.1111/j.1365-2982.2004.00549.x 15601431

[B39] Massa F Sibaev A Marsicano G Blaudzun H Storr M Lutz B Vanilloid receptor (TRPV1)-deficient mice show increased susceptibility to dinitrobenzene sulfonic acid induced colitis J Mol Med (Berl) 2006 84 142 146 10.1007/s00109-005-0016-2 16389550

[B40] Szitter I Pozsgai G Sandor K Elekes K Kemeny A Perkecz A Szolcsanyi J Helyes Z Pinter E The role of transient receptor potential vanilloid 1 (TRPV1) receptors in dextran sulfate-induced colitis in mice J Mol Neurosci 2010 42 80 88 10.1007/s12031-010-9366-5 20411352

[B41] Banvolgyi A Pozsgai G Brain SD Helyes ZS Szolcsanyi J Ghosh M Melegh B Pinter E Mustard oil induces a transient receptor potential vanilloid 1 receptor-independent neurogenic inflammation and a non-neurogenic cellular inflammatory component in mice Neuroscience 2004 125 449 459 10.1016/j.neuroscience.2004.01.009 15062987

[B42] Birder LA Nakamura Y Kiss S Nealen ML Barrick S Kanai AJ Wang E Ruiz G de Groat WC Apodaca G Watkins S Caterina MJ Altered urinary bladder function in mice lacking the vanilloid receptor TRPV1 Nat Neurosci 2002 5 856 860 10.1038/nn902 12161756

[B43] Daly D Rong W Chess-Williams R Chapple C Grundy D Bladder afferent sensitivity in wild-type and TRPV1 knockout mice J Physiol 2007 583 663 674 10.1113/jphysiol.2007.139147 17627983 PMC2277033

[B44] Charrua A Cruz CD Cruz F Avelino A Transient receptor potential vanilloid subfamily 1 is essential for the generation of noxious bladder input and bladder overactivity in cystitis J Urol 2007 177 1537 1541 10.1016/j.juro.2006.11.046 17382774

[B45] Dinis P Charrua A Avelino A Yaqoob M Bevan S Nagy I Cruz F Anandamide-evoked activation of vanilloid receptor 1 contributes to the development of bladder hyperreflexia and nociceptive transmission to spinal dorsal horn neurons in cystitis J Neurosci 2004 24 11253 11263 10.1523/JNEUROSCI.2657-04.2004 15601931 PMC6730374

[B46] Wang ZY Wang P Merriam FV Bjorling DE Lack of TRPV1 inhibits cystitis-induced increased mechanical sensitivity in mice Pain 2008 139 158 167 10.1016/j.pain.2008.03.020 18445509

[B47] Yu W Hill WG Apodaca G Zeidel ML Expression and distribution of transient receptor potential (TRP) channels in bladder epithelium Am J Physiol Renal Physiol 2011 300 F49 F59 10.1152/ajprenal.00349.2010 20943764 PMC3023226

[B48] Caterina MJ Vanilloid receptors take a TRP beyond the sensory afferent Pain 2003 105 5 9 10.1016/S0304-3959(03)00259-8 14499414

[B49] Xu YP Zhang JW Li L Ye ZY Zhang Y Gao X Li F Yan XS Liu ZG Liu LJ Cao XH Complex regulation of capsaicin on intracellular second messengers by calcium dependent and independent mechanisms in primary sensory neurons Neurosci Lett 2012 517 30 35 10.1016/j.neulet.2012.04.011 22516465

[B50] Mandadi S Armati PJ Roufogalis BD Protein kinase C modulation of thermo-sensitive transient receptor potential channels: Implications for pain signaling J Nat Sci Biol Med 2011 2 13 25 10.4103/0976-9668.82311 22470230 PMC3312694

[B51] Studer M McNaughton PA Modulation of single-channel properties of TRPV1 by phosphorylation J Physiol 2010 588 3743 3756 10.1113/jphysiol.2010.190611 20693293 PMC2998224

[B52] Zhang X Disanto ME Rho-kinase, a common final path of various contractile bladder and ureter stimuli Handb Exp Pharmacol 2011 202 543 568 10.1007/978-3-642-16499-6_24 21290242

[B53] Sward K Dreja K Susnjar M Hellstrand P Hartshorne DJ Walsh MP Inhibition of Rho-associated kinase blocks agonist-induced Ca2+ sensitization of myosin phosphorylation and force in guinea-pig ileum J Physiol 2000 522 Pt 1 33 49 10618150 10.1111/j.1469-7793.2000.0033m.x PMC2269742

[B54] Janssen LJ Tazzeo T Zuo J Pertens E Keshavjee S KCl evokes contraction of airway smooth muscle via activation of RhoA and Rho-kinase Am J Physiol Lung Cell Mol Physiol 2004 287 L852 L858 10.1152/ajplung.00130.2004 15208091

[B55] Mita M Yanagihara H Hishinuma S Saito M Walsh MP Membrane depolarization-induced contraction of rat caudal arterial smooth muscle involves Rho-associated kinase Biochem J 2002 364 431 440 10.1042/BJ20020191 12023886 PMC1222588

[B56] Urban NH Berg KM Ratz PH K+ depolarization induces RhoA kinase translocation to caveolae and Ca2+ sensitization of arterial muscle Am J Physiol Cell Physiol 2003 285 C1377 C1385 12890649 10.1152/ajpcell.00501.2002

[B57] Jezior JR Brady JD Rosenstein DI McCammon KA Miner AS Ratz PH Dependency of detrusor contractions on calcium sensitization and calcium entry through LOE-908-sensitive channels Br J Pharmacol 2001 134 78 87 10.1038/sj.bjp.0704241 11522599 PMC1572931

[B58] Zhu ZY Zhang LL Wang PJ Ma LQ Wang LJ Liu DY Zhu ZM Activation of transient receptor potential vanilloid 1 inhibits RhoA/Rho kinase and improves vasorelaxation dysfunction mediated by high-fat diet in mice Zhongguo Yi Xue Ke Xue Yuan Xue Bao 2011 33 600 605 22509539

[B59] Fujimoto S Mori M Characterization of capsaicin-induced, capsazepine-insensitive relaxation of ileal smooth muscle of rats Eur J Pharmacol 2004 487 175 182 10.1016/j.ejphar.2004.01.014 15033390

[B60] Maggi CA The dual function of capsaicin-sensitive sensory nerves in the bladder and urethra Ciba Found Symp 1990 151 77 83 2226067 10.1002/9780470513941.ch5

[B61] Philyppov IB Paduraru ON Andreev YA Grishin EV Shuba YM Modulation of TRPV1-dependent contractility of normal and diabetic bladder smooth muscle by analgesic toxins from sea anemone Heteractis crispa Life Sci 2012 91 912 920 10.1016/j.lfs.2012.09.001 22982418

[B62] Ho C O'Leary ME Single-cell analysis of sodium channel expression in dorsal root ganglion neurons Mol Cell Neurosci 2011 46 159 166 10.1016/j.mcn.2010.08.017 20816971 PMC3005531

[B63] Waxman SG Sodium channels, the electrogenisome and the electrogenistat: lessons and questions from the clinic J Physiol 2012 590 2601 2612 10.1113/jphysiol.2012.228460 22411010 PMC3424719

[B64] Prinz AA Abbott LF Marder E The dynamic clamp comes of age Trends Neurosci 2004 27 218 224 10.1016/j.tins.2004.02.004 15046881

[B65] MacLean JN Zhang Y Johnson BR Harris-Warrick RM Activity-independent homeostasis in rhythmically active neurons Neuron 2003 37 109 120 10.1016/S0896-6273(02)01104-2 12526777

[B66] Swensen AM Bean BP Robustness of burst firing in dissociated purkinje neurons with acute or long-term reductions in sodium conductance J Neurosci 2005 25 3509 3520 10.1523/JNEUROSCI.3929-04.2005 15814781 PMC6725377

[B67] Beyak MJ Ramji N Krol KM Kawaja MD Vanner SJ Two TTX-resistant Na+ currents in mouse colonic dorsal root ganglia neurons and their role in colitis-induced hyperexcitability Am J Physiol Gastrointest Liver Physiol 2004 287 G845 G855 10.1152/ajpgi.00154.2004 15205116

[B68] Lamb K Zhong F Gebhart GF Bielefeldt K Experimental colitis in mice and sensitization of converging visceral and somatic afferent pathways Am J Physiol Gastrointest Liver Physiol 2006 290 G451 G457 10.1152/ajpgi.00353.2005 16195421

[B69] Chen TY Corcos J Camel M Ponsot Y Tu lM Prospective, randomized, double-blind study of safety and tolerability of intravesical resiniferatoxin (RTX) in interstitial cystitis (IC) Int Urogynecol J Pelvic Floor Dysfunct 2005 16 293 297 10.1007/s00192-005-1307-4 15818465

[B70] Sculptoreanu A Kullmann FA Artim DE Bazley FA Schopfer F Woodcock S Freeman BA de Groat WC Nitro-oleic acid inhibits firing and activates TRPV1- and TRPA1-mediated inward currents in dorsal root ganglion neurons from adult male rats J Pharmacol Exp Ther 2010 333 883 895 10.1124/jpet.109.163154 20304940 PMC2879937

[B71] Cao X Cao X Xie H Yang R Lei G Li F Li A Liu C Liu L Effects of capsaicin on VGSCs in TRPV1−/− mice Brain Res 2007 1163 33 43 17632091 10.1016/j.brainres.2007.04.085

[B72] Bautista DM Jordt SE Nikai T Tsuruda PR Read AJ Poblete J Yamoah EN Basbaum AI Julius D TRPA1 mediates the inflammatory actions of environmental irritants and proalgesic agents Cell 2006 124 1269 1282 10.1016/j.cell.2006.02.023 16564016

[B73] Staruschenko A Jeske NA Akopian AN Contribution of TRPV1-TRPA1 interaction to the single channel properties of the TRPA1 channel J Biol Chem 2010 285 15167 15177 10.1074/jbc.M110.106153 20231274 PMC2865321

[B74] Ohta T Imagawa T Ito S Novel gating and sensitizing mechanism of capsaicin receptor (TRPV1): tonic inhibitory regulation of extracellular sodium through the external protonation sites on TRPV1 J Biol Chem 2008 283 9377 9387 10.1074/jbc.M709377200 18230619

[B75] Laird JM Martinez-Caro L Garcia-Nicas E Cervero F A new model of visceral pain and referred hyperalgesia in the mouse Pain 2001 92 335 342 10.1016/S0304-3959(01)00275-5 11376906

[B76] Toth DM Szoke E Bolcskei K Kvell K Bender B Bosze Z Szolcsanyi J Sandor Z Nociception, neurogenic inflammation and thermoregulation in TRPV1 knockdown transgenic mice Cell Mol Life Sci 2011 68 2589 2601 10.1007/s00018-010-0569-2 21069423 PMC11115187

[B77] Christoph T Grunweller A Mika J Schafer MK Wade EJ Weihe E Erdmann VA Frank R Gillen C Kurreck J Silencing of vanilloid receptor TRPV1 by RNAi reduces neuropathic and visceral pain in vivo Biochem Biophys Res Commun 2006 350 238 243 10.1016/j.bbrc.2006.09.037 16996476

[B78] Honore P Wismer CT Mikusa J Zhu CZ Zhong C Gauvin DM Gomtsyan A El KR Lee CH Marsh K Sullivan JP Faltynek CR Jarvis MF A-425619 [1-isoquinolin-5-yl-3-(4-trifluoromethyl-benzyl)-urea], a novel transient receptor potential type V1 receptor antagonist, relieves pathophysiological pain associated with inflammation and tissue injury in rats J Pharmacol Exp Ther 2005 314 410 421 10.1124/jpet.105.083915 15837818

[B79] Caterina MJ Leffler A Malmberg AB Martin WJ Trafton J Petersen-Zeitz KR Koltzenburg M Basbaum AI Julius D Impaired nociception and pain sensation in mice lacking the capsaicin receptor Science 2000 288 306 313 10.1126/science.288.5464.306 10764638

[B80] Christoph T Bahrenberg G De VJ Englberger W Erdmann VA Frech M Kogel B Rohl T Schiene K Schroder W Seibler J Kurreck J Investigation of TRPV1 loss-of-function phenotypes in transgenic shRNA expressing and knockout mice Mol Cell Neurosci 2008 37 579 589 10.1016/j.mcn.2007.12.006 18249134

[B81] Kasama S Kawakubo M Suzuki T Nishizawa T Ishida A Nakayama J RNA interference-mediated knock-down of transient receptor potential vanilloid 1 prevents forepaw inflammatory hyperalgesia in rat Eur J Neurosci 2007 25 2956 2963 10.1111/j.1460-9568.2007.05584.x 17509082

